# 599 Eyelash Reconstruction Utilizing a Strip Graft for Burn-related Madarosis: A Case Report and Operative Technique

**DOI:** 10.1093/jbcr/irac012.227

**Published:** 2022-03-23

**Authors:** Daniel N Driscoll, Robert J Dabek

**Affiliations:** Massachusetts General Hospital, Boston, Massachusetts; Ascension St.Agnes Hospital, Baltimore, Maryland

## Abstract

**Introduction:**

Facial burns are a common occurrence with a small percentage causing injury to the periocular region. Although the eye itself is often spared, madarosis may occur from deep burn injury to the eyelid. Reconstruction of the eyelid margin is technically difficult due to the challenging aesthetic region, as well as specific characteristics of eyelashes. Although there are several options for eyelash grafting there is no established gold standard, and there is little to no-literature related to procedures involving burn madarosis.

**Methods:**

Here we describe a case of unilateral eyelash grafting utilizing a composite strip graft in a 20-year-old female who sustained a 70% flame injury at the age of two.

**Results:**

The presented results show the efficacy of this technique at restoring function and providing a good aesthetic result which is maintained at 1 year follow up.

**Conclusions:**

Utilization of a composite strip graft to recreate the eyelid margin is a viable choice for reconstruction of burned upper eyelids in the appropriate patient. Further exploration regarding procedure timing and choice of donor site are warranted.

